# Functional MRI in Awake Dogs Predicts Suitability for Assistance Work

**DOI:** 10.1038/srep43704

**Published:** 2017-03-07

**Authors:** Gregory S. Berns, Andrew M. Brooks, Mark Spivak, Kerinne Levy

**Affiliations:** 1 Psychology Department, Emory University, Atlanta, GA, USA; 2 Dog Star Technologies, Sandy Springs, GA, USA; 3 Canine Companions for Independence, Santa Rosa, CA, USA.

## Abstract

The overall goal of this work was to measure the efficacy of fMRI for predicting whether a dog would be a successful service dog. The training and imaging were performed in 49 dogs entering service training at 17–21 months of age. 33 dogs completed service training and were matched with a person, while 10 were released for behavioral reasons (4 were selected as breeders and 2 were released for medical reasons.) After 2 months of training, fMRI responses were measured while each dog observed hand signals indicating either reward or no reward and given by both a familiar handler and a stranger. Using anatomically defined ROIs in the caudate, amygdala, and visual cortex, we developed a classifier based on the dogs’ subsequent training outcomes. The classifier had a positive predictive value of 94% and a negative predictive value of 67%. The area under the ROC curve was 0.91 (0.80 with 4-fold cross-validation, P = 0.01), indicating a significant predictive capability. The magnitude of response in the caudate was positively correlated with a successful outcome, while the response in the amygdala depended on the interaction with the visual cortex during the stranger condition and was negatively correlated with outcome (higher being associated with failure). These results suggest that, as indexed by caudate activity, successful service dogs generalize associations to hand signals regardless who gives them but without excessive arousal as measured in the amygdala.

The advent of awake fMRI in dogs has opened up numerous possibilities for decoding how the dog’s brain is organized^1^,^2^,^3^. But like many of the early human fMRI studies, these nascent efforts have been plagued by small sample sizes. Some results have been replicated, increasing confidence in the technique^4^,^5^,^6^. At the same time, there have also been hints of substantial individual differences between dogs’ brain responses that, like humans, relate to important aspects of behavior, temperament, and personality^7^,^8^.

We have previously observed a temperament-dependent increase in neural activity in the caudate when dogs are presented with a hand-signal associated with incipient receipt of food reward^8^. Dogs who came from a service-dog training program were more likely to show a caudate response to a hand signal when interacting with their owner/handler, while other dogs (e.g. from shelters and pets with no service-training) were more likely to show a caudate response to the signal when interacting with an unfamiliar person.

Are these differences a result of service-training, or might there be a neurobiological phenotype that contributes to a dog’s ability to perform tasks required of service dogs? If the latter, then it should be possible to identify attributes and neurobiological correlates that predict successful completion of service-training. Most dogs are not suited to be service dogs. Although there is no industry standard, a well-managed service-dog program considers heredity in breeding, optimal nutrition during gestation and nursing, neonatal care of pups and nutrition after weaning, maintenance of hygienic environments, and provision of socialization opportunities with other dogs, animals, and people. Even so, up to 70% of dogs from such programs are deemed unsuitable for service work. By many estimates, the cost of training a service dog is $20,000 to $50,000. If dogs that are predisposed to fail training could be identified earlier, the average cost would decline.

There have been several studies of juvenile dogs, using behavioral tests and questionnaire ratings, that have examined the possibility of identifying high-potential working dogs. The earliest studies of puppy temperament indicated some trait consistency, but not to the extent of a predictive test^9^. Later studies showed that working dogs, on average, had different behavioral traits but these were not consistent enough to predict individual performance as adults^10^,^11^,^12^. As dogs get older, their behavior becomes more consistent, achieving some level of stability between 12 and 24 months of age, to the point where temperament questions and behavioral tests become modestly predictive of suitability for service work^13^,^14^. However, the variability of inter-rater agreement and test-retest reliability has raised questions about the utility of these approaches^15^,^16^.

To determine if a pattern of brain responses can predict completion of training and placement in a service job, we performed a prospective fMRI study of 49 dogs at the beginning of their service-training. We focused on three brain regions as potential biomarkers of success or failure: 1) caudate for reward sensitivity^4^,^17^; 2) amygdala for arousal^18^; and 3) a region of temporal cortex previously shown to be responsive to faces^5^.

## Methods

### Participants

All dogs participating in the study came from Canine Companions for Independence (CCI, Santa Rosa, CA). CCI’s dogs undergo a controlled socialization process. After they are weaned, puppies are raised by a volunteer puppy-raiser until 17–21 months of age. Then, the dogs are returned to one of CCI’s training facilities for service training, which can take 6–9 months. Dogs that complete the training “graduate” into one of several roles: 1) service dog (assists adults with physical disabilities with daily activities); 2) skilled companion (assists adults and children under the guidance of a facilitator, e.g. parent or spouse); 3) facility dog (works with a facilitator in an institutional setting, e.g. hospital); 4) hearing dog (trained to recognize specific sounds and alert the person); and more recently, 5) PTSD dog (provides social buffer, conducts room searches and provides “all-clear” signal). Those who are unable to complete training, for either medical or behavioral reasons, are “released” and adopted, often by the puppy-raiser. The study was approved by both the Emory Institutional Animal Care and Use Committee and the UC Berkeley Animal Care and Use Committee. All methods and protocols were carried out in accordance with federal regulations under the Animal Welfare Act.

Between 11/2014 and 11/2015, 54 dogs were selected for participation in the MRI training program. The selection occurred within two weeks of beginning service training. (The exception was the initial dog to train in the protocol, who had already worked as a service dog and was retired.) Dogs were first assessed for absence of noise reactivity by playing recordings of the scanner noise, and then 2–3 dogs were randomly picked from each trainer’s string for participation in additional MRI training. For consistency, the MRI training was conducted by three trainers throughout the project. Cohorts of 6–12 dogs were selected every 3 months, until the target of 50 was reached (4 dogs did not complete the training). One dog completed training, but did not successfully complete the MRI session by repeatedly exiting the scanner. Four females were selected for breeding, and thus, no training outcome was obtained. Two dogs were released for medical reasons. This left a total of 43 dogs for which both MRI data and outcomes were obtained. The dogs were predominately crosses of Labrador retrievers and golden retrievers, with a few purebred Labs and goldens (Table 1).

### Training

**Figure 1:**
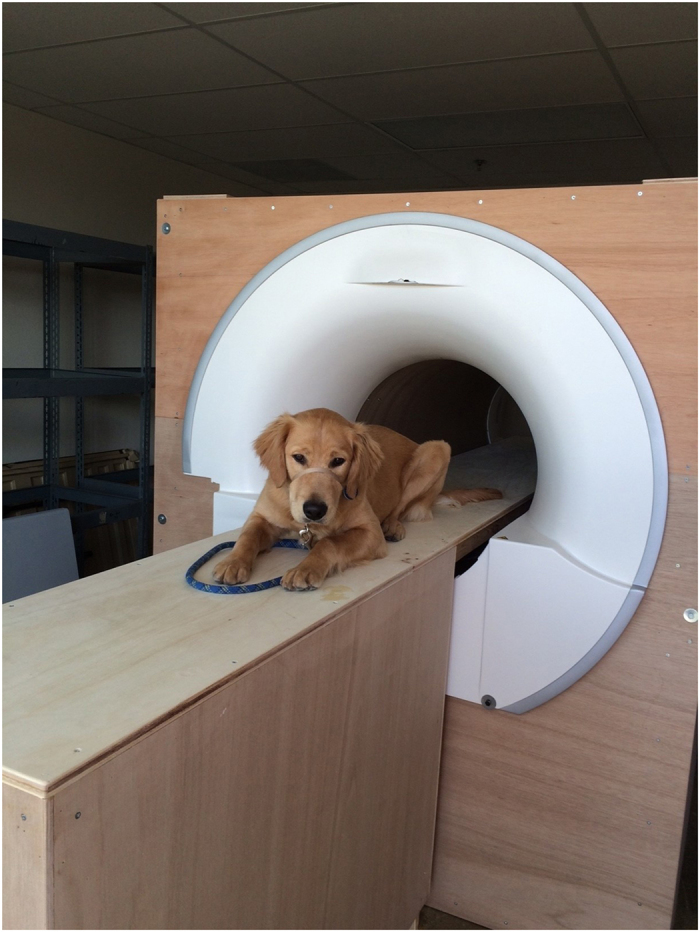
Mock scanner. A dog is shown on the patient table. Speakers to simulate the acoustic noise are inside the simulator.

MRI-training took place on the CCI campus in Santa Rosa, CA. A mock-MRI was constructed on site from discarded parts of a Siemens Trio. The mock-MRI included a patient table, bore, replicas of head and neck coils, and loudspeakers to play the noises from the scan sequences (Fig. 1). The training took approximately 10–15 minutes per dog, three days a week. All dogs were judged MRI-ready after two months.

The training program teaches the dogs to cooperatively enter the MRI scanner. The program is based on acclimatization to the MRI scanner noise, tight scanner enclosure, scanner steps, and operating vibrations and the shaping and ultimate chaining of several requisite behaviors. We also constructed a customized chin rest that facilitated comfort and proper positioning for the dogs and that adapted the apparatus for the uniqueness of the canine anatomy. Once the animals became confident and competent regarding all the preparatory steps – proven by completing a simulated MRI in the replica apparatus – we then performed live scans in the actual MRI at UC Berkeley.

We compiled digitized audio recordings of the various scanner sequences. To aid in the necessary desensitization and acclimation to the scanner noises, when training, we played the recordings through a portable speaker placed inside the simulator.

Only positive reinforcement, in combination with behavioral shaping, conditioning and chaining, were used in the training process. First, dogs were trained to place their head and paws in the head coil. Next, they were trained to place their chin on a chin rest placed horizontally across the head coil and hold this position until a release signal. The length of the hold was gradually increased up to 30 s. When the dogs were able to do this consistently with no discernible head motion, they were next trained to do this wearing ear plugs and vet wrap, which were initially introduced to the animals apart from the coil simulator. Concurrent with the initial sequences of the training, recordings of the scanner noise were introduced at low volume. Once the animal became conditioned at a low volume, the volume was gradually increased. Recordings of the scanner noise were introduced at low volume while the dog remained stationary in the coil. Once the dog demonstrated relaxed behavior, the volume was gradually increased. When the dogs were comfortable wearing the ear plugs in the head coil with the scanner noise of approximately 90 dB, they were then trained to go into the MRI tube. Subsequently, the simulated head coil was placed inside the tube. After the dog was consistently holding her head still in this configuration, the entire apparatus was raised on a table to the height of the actual scanner patient table. The dogs were trained to walk up steps into the mock-MRI.

### Imaging

The MRI protocol is similar to that previously described^1^,^4^. We have found that the neck coil that is standard on a Siemens Trio is well suited to scanning dogs’ brains while in a sphinx position. The chin rest was constructed from firm foam, and semicircles were cut to match the shape of the dog’s muzzle from the nose to the ramus of the mandible. The chin rest was inserted within the inner diameter of the coil during scanning.

When performing an actual scan, immediately prior to the scan, audio recordings of the pertinent scan sequence were played through a speaker in the magnet room. As the dog settled in the scanner, the volume was increased to match the decibel level of the actual scanner noise. While playing a continuous loop of the recording, the actual scan was begun. The recordings were effective at minimizing the startle response that would otherwise result from the sudden onset of the real scan. Once the scan actual began, the scanner recording was turned off.

First, a single sagittal plane image was acquired as a localizer, which lasted 3 s (SPGR sequence, slice thickness = 4 mm. TR = 9.2 ms, TE = 4.16 ms, flip angle = 40°, 256 × 256 matrix, FOV = 220mm). The localizer sound tended to be the most startling and unpleasant for the dogs. This was minimized by acquiring a single plane, but repeating it 3 times in case the dog startled at the onset. Because the chin rest centered the dog in the left-right direction, a single sagittal image was all that was necessary for planning the field-of-view for the subsequent scans.

After the localizer, a T2-weighted structural image was acquired with a turbo spin-echo sequence (28 2 mm slices, TR = 3500 ms, TE = 11 ms, flip angle = 131°, turbo factor = 15, 128 × 128 matrix, 1.5 × 1.5 mm in-plane resolution), which lasted ~30 s. We used low-SAR and whisper mode to minimize acoustic noise. This sequence was optimized to yield good contrast between gray and white matter in the fastest time possible.

For functional scans, we used single-shot echo-planar imaging (EPI) to acquire volumes of 21 sequential 2.5 mm slices with a 20% gap (TE = 25 ms, TR = 1200 ms, flip angle = 70°, 70 × 70 matrix, FOV = 208 mm, 3 × 3 mm in-plane resolution). Slices were oriented dorsally to the dog’s brain (coronal to the magnet because the dog was positioned 90° from the usual human orientation) with the phase-encoding direction right-to-left. Sequential scans minimized between-plane offsets when the dog moved. The 20% slice gap minimized crosstalk for sequential acquisitions. The right-left phase encoding minimized ghost images from the neck that would otherwise overlap into the dog’s brain. TE was decreased slightly to minimize distortion from the frontal sinus. TR was as short as possible to acquire enough slices to cover the entire brain while not so short as to significantly decrease signal.

### Experimental Design

**Figure 2:**
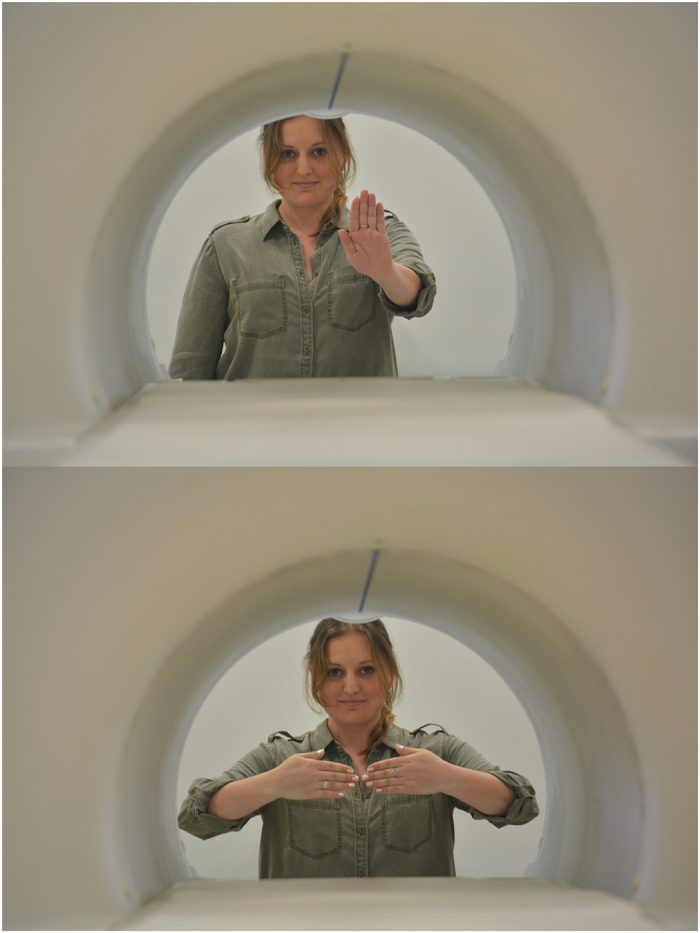
Hand signals. “Reward” hand signal (*top*) and “no reward” signal (*bottom*).

The imaging protocol was an adaption of a previous experiment^8^. During training, dogs were taught two hand signals: one representing a food treat (reward) and one representing no treat (no reward) (Fig. 2). Each hand signal was shown for 10 s, allowing enough time for the HRF to reach a peak before the dog moved to eat the treat. The key manipulation was who gave the signal: handler with whom the dog has been training or a stranger. There were four functional runs (each with 15 reward trials and 15 no-reward trials): 1) handler; 2) stranger; 3) stranger; 4) handler. The order of runs was counterbalanced in time to avoid confounding nonspecific effects due to repetition. Depending on the speed of the human, each run lasted 5–7 minutes, yielding 250–350 volumes/run, for a total 1000–1500 volumes. With time for acclimation and breaks between scans, the entire session lasted about 1 hour for each dog.

### Data Analysis

#### Preprocessing

Functional data were preprocessed using AFNI and its associated functions. DICOM files of the EPI runs were first converted to AFNI BRIK format using the to3d command. The EPI runs were then subjected to motion correction using 3dvolreg’s 6-parameter affine transformation, employing a two-pass method, where the first pass results in a crude alignment and the second pass a fine alignment. All volumes were aligned to a reference volume, which was manually chosen volume from the first run based on a visual inspection.

Because of the dog’s intermittent motion, censoring (removal) of volumes with artifacts is crucial. We used three separate methods to censor volumes with motion artifacts. First, 3dToutcount was used to output the fraction of outlier voxels for each volume. 3dToutcount defines outliers as those voxels whose signal intensity deviates from the median absolute deviation of the time series. Volumes with a fraction larger than 0.01 were censored from the statistical analysis. Second, 1d_tool.py was used to censor volumes based on the amount of estimated motion outputted from 3dvolreg. 1d_tool.py computes the Euclidean norm of the derivative of the rotation and translation parameters outputted from 3dvolreg. We then used a Euclidean norm cut-off of 1 to generate the censor file. Finally, we visually inspected the resulting time series with the censored volumes from 3dToutcount and 1d_tool.py, and censored any volumes that still showed obvious artifact. The majority of the censored volumes followed the consumption of the food reward.

The EPI images were then smoothed and normalized to %-signal change. Smoothing was applied using 3dmerge, with a 6 mm kernel at Full-Width Half-Maximum (FWHM). To convert signal intensity values to %-signal change, 3dcalc was used to subtract and then divide by the mean EPI image. These values were then converted to percentages by multiplying by 100.

#### General Linear Model (GLM)

For each subject, a GLM was estimated for each voxel using 3dDeconvolve. The task-related regressors included: 1) handler reward hand signal (Rew_hand_); 2) handler no-reward hand signal (NoRew_hand_); 3) stranger reward hand signal (Rew_str_); and 4) stranger no-reward hand signal (NoRew_str_). All events were specified as variable duration events using the dmUBLOCK function. To control for subject movement, the 6 motion regressors output from 3dvolreg were also included in the model. To account for differences between runs, a constant and linear drift term were included for each run. We generated 2 contrasts that depended on the source of the hand signals: 1) Handler: [Rew_hand _− NoRew_hand_]; and 2) Stranger: [Rew_str_ − NoRew_str_].

#### Spatial Normalization

For each dog, three spatial transformations were computed: 1) mean EPI to structural (affine); 2) structural to template (affine); and 3) diffeomorphic structural to template^19^. Images were manually skull-stripped prior to computing these transformations. The transformations were computed using the software package, Advanced Normalization Tools (ANTs)^20^. In all cases, the global correlation metric was used with 3 iteration levels at progressively finer resolution. The diffeomorphic transformation to the template image used the symmetric normalization (SyN) model with 3 iteration levels. These transformations were concatenated together and applied to individual contrasts obtained from the statistical model described above as well as the structural image and mean EPI for each dog. The end result was an image for each dog transformed into template space, allowing the computation of a group level statistic across all dogs and extraction of ROI values.

**Figure 3:**
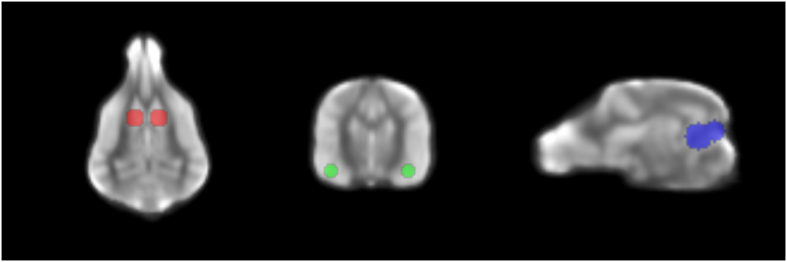
Regions of interest. ROIs are overlaid on the mean structural image for all dogs after spatial normalization: caudate (*red*), amygdala (*green*), and dog face area (DFA) of visual cortex (*blue*).

The transformed structural and mean EPI images were checked for good co-registration to each other and to the template image. Because of the oblong shape of the dog’s brain, and the long olfactory bulbs, there is the potential for different degrees of normalization in each region. To check the overall consistency, we computed an average across all dogs of the transformed structural images in template space and used this for ROI placement (Fig. 3). Variations in brain size and morphology can affect the normilazation procedure and subsequent ROI extractions, but because the dogs were all varying degrees of Labrador and golden retriever, their brain size and morphology was more similar than in previous fMRI studies across multiple breeds. As a result, the normalization was very consistent.

#### Regions-of-interest (ROIs)

Based on previous research, we defined 3 ROIs (Fig. 3): 1) bilateral caudate (CD); 2) bilateral amygdala (AMY); and 3) a region of visual cortex/temporal lobe previously identified to be face-selective in dogs, called dog face area (DFA)^5^. The amygdala ROI was based on the assumption that activity there would be related to arousal and could be relevant for predicting service dog success. The DFA was based on the possibility that differences in face-processing might also be relevant. The caudate and amygdala ROIs were spheres centered on these structures in template space. The DFA ROI was oblong and based on the location previously observed (ROI values for all dogs and conditions is in supplementary data).

#### Classifier

The primary goal of the project was to develop and test a brain-based classification algorithm that would predict successful graduation of service dogs. We used both the scikit-learn (http://scikit-learn.org) package in Python and glmfit function in Matlab to perform model-fitting, feature selection, and cross-validation. First, we performed feature selection by fitting the ROI data and two demographic parameters: sex and responsiveness. The responsiveness score was assigned by the dogs’ trainers mid-way through formal training based on the dog’s overall temperament, around the time of MRI-scanning. Values ranged from 1–6, with a higher score being more likely to succeed in the program and indicated a dog that is focused on and responsive to its handler. Responsiveness scores were assigned by handlers unaffiliated with the actual MRI training.

We tested several kernels including logistic, naïve Bayes, and linear support vector machine (SVM). We found that logistic regression consistently performed the best and so used that for feature selection. Because of the imbalance in dog outcomes, failures were weighted 3.3 times successes in the regression. First, we evaluated a behavioral-only model that consisted of sex and responsiveness score. This model is important because it provides a metric of performance that could be obtained without the need for brain-imaging. To be of practical use, a brain-based model should perform at least as well as this, and ideally, improve the performance of the classifier when combined with conventional behavioral measures. For feature selection, we tested the following models: 1) responsiveness; 2) sex + responsiveness; 3) sex + 4 ROIs; 4) responsiveness + 4 ROIs; 5) sex + responsiveness + 4 ROIs; and 6) sex + breed + 4 ROIs. For example, model #3 was defined:


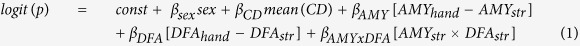


where:


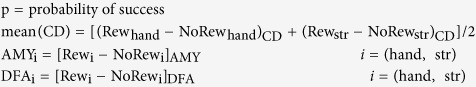


Thus, this model used the differential activity in the caudate to reward and no-reward hand signals averaged over both the handler and stranger conditions, the differential between reward signals and source (handler vs. stranger) in the amygdala and DFA, and the interaction between amygdala and DFA in the stranger condition.

We calculated confusion matrices for each model with a threshold of 0.5, but for overall comparisons, we used the area-under-the curve (AUC) for the receiver operating characteristic (ROC), which plots true positive rate (TPR) vs. false positive rate (FPR) and thus does not depend on a particular threshold.

## Results

The MRI-dogs had a high proportion of successes with 77% being placed in one of the assistance-dog categories (Table 2). Only 10 dogs were released for behavioral reasons. Sex was not a statistically significant factor (Table 3).

**Figure 4:**
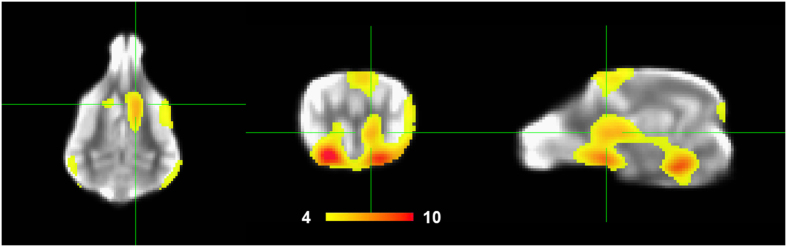
Average activation of [reward – no reward] hand signals. Contrast is averaged across both the handler and stranger conditions and thresholded at p < 10^−4^. Color scale runs from T = 4 to T = 10. Robust bilateral activation of caudate is seen.

As a check that the experiment was broadly consistent with previous imaging results, we first examined the whole-brain contrast for [reward – no reward] hand signals, averaged across all runs (both handler and stranger). This resulted in robust activation of the striatum (Fig. 4), confirming the validity of the paradigm.

**Figure 5:**
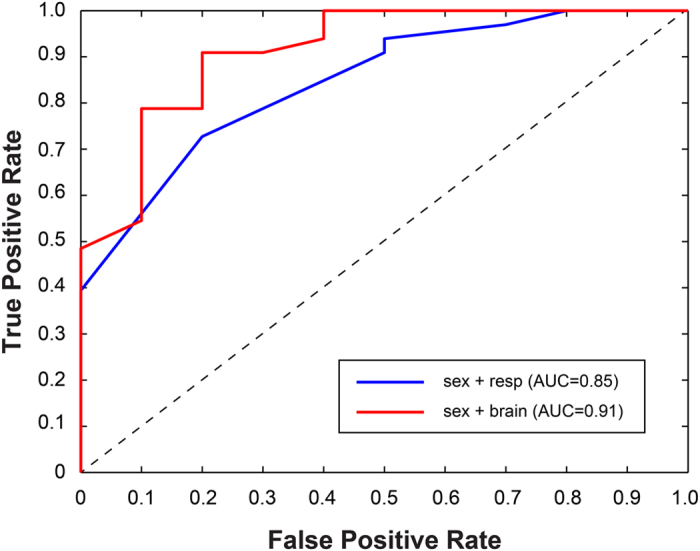
ROC plot of 2 classifier models. The combination of sex and responsiveness (*blue*) is a model that could be done without fMRI data (AUC = 0.85). The combination of sex, mean caudate activity, differential amygdala and DFA, plus interaction in the stranger condition (*red*) performed the best (AUC = 0.91).

Both the behavior-only classifiers performed above chance. The sex + responsiveness model did well, achieving an AUC = 0.85 (Fig. 5, *blue*). However, the sex + 4 ROIs model (Eq. 1) did better, achieving an AUC = 0.91 (Fig. 5, *red*). Including responsiveness increased the AUC to 0.94, but that term was not significant with the ROIs. Similarly, breed was a non-significant effect. The confusion matrices showed that the brain model did better at classifying failures (Table 4). Moreover, the signs of the coefficients are interpretable (Table 5). The mean differential response to [reward – no reward] was positively correlated to success in the caudate, while the interaction between the amygdala and the DFA in the stranger condition was negatively correlated with success.

**Figure 6:**
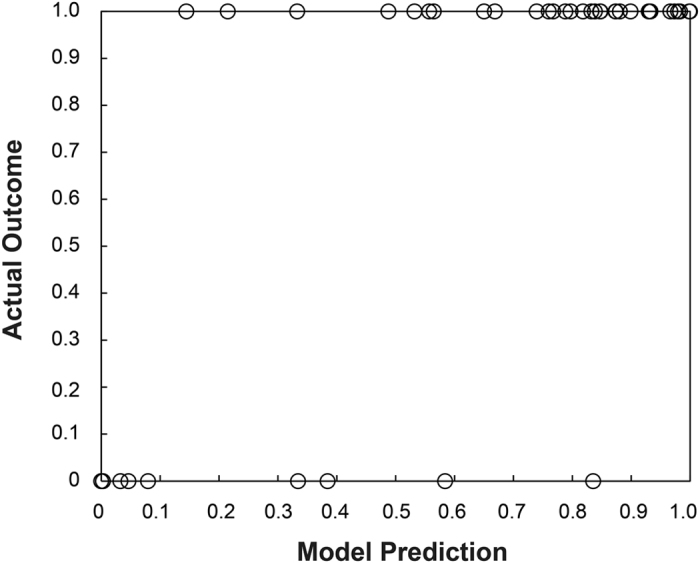
Model performance. Performance of the sex + brain model vs. actual outcome. 0 = fail; 1 = success.

**Figure 7:**
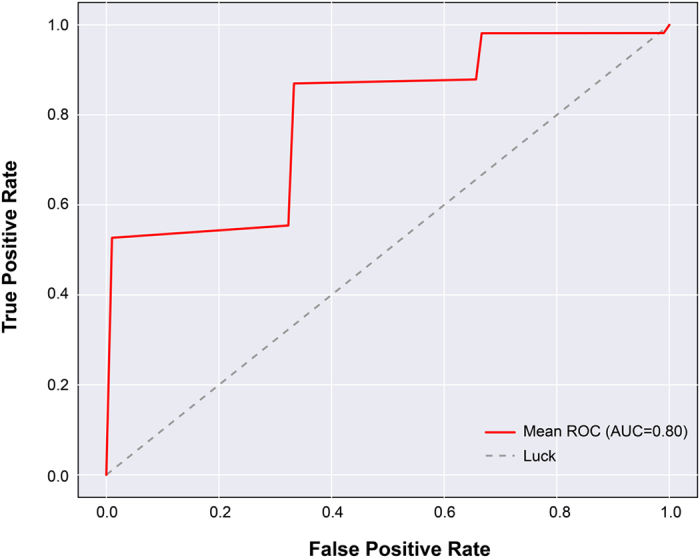
Cross-validated model. Estimate of real-world performance of the sex + brain model using 4-fold cross-validation (AUC = 0.80, P = 0.01).

Because these models were fit to all of the data, the performance represents a best-case scenario and would not be expected to do this well with new data (Fig. 6). To get a closer estimate of real-world performance, we performed the same classification of the final model using 1000 iterations of stratified random shuffling, with a test size of 25% (4 folds). Stratified random shuffling balances successes and failures for each split, which is important given the high class imbalance. We chose a 75/25 train/test split on each iteration due to the small sample size that would otherwise lead to high variance in accuracy estimates if smaller training sizes were used. As expected, the performance was not as good as the model fit to all of the data, but the AUC = 0.80, which is still considered moderately good (Fig. 7).

## Discussion

The primary goal of this study was to determine the efficacy of awake dog fMRI in predicting a dog’s suitability for assistance work. Using a paradigm in which the dog passively responded to hand signals indicating incipient food reward or nothing, we found that the person giving the signals affected the dogs’ brain responses. In particular, when a stranger gave the signals, relative to a familiar handler, we could use the differential activation in three regions-of-interest to predict the likelihood of a dog succeeding in a particular program of assistance training.

The brain-imaging model performed better (AUC = 0.91) than the sex + behavior model (AUC = 0.85), suggesting that brain activations may contain information about internal states that would otherwise be unobservable from behavior alone. This alone may not warrant the use of brain imaging as a screening assessment for potential service dogs when common demographics and behavior tests do almost as well. These levels of predictive value are comparable to that reported in previous studies of behavioral tests of juvenile dogs^13^. However, combining brain activation with sex and behavior resulted in an improvement in predictive power, increasing the AUC to 0.94. Depending on the threshold chosen, this resulted in a PPV of up to 94% (Table 4). The brain data seem to be particularly valuable in decreasing the false negatives at any given threshold.

Although the results reported here represent the largest single fMRI study of dogs, the sample size is still considered small for predictive modeling. When the study was designed, we anticipated a 60% failure rate. Instead, we observed a 25% failure rate, which limits the generalizability of the model results. To mitigate the possibility of overfitting the model to the data, we performed cross-validation using stratified random shuffling. Stratification preserves the ratio of outcomes in each train/test iteration. With such a small sample size, we run the risk of noise dominating any individual iteration and so this type of validation is likely overly conservative, but this may counteract the optimistic performance obtained from the full dataset. The cross-validation suggested an AUC of 0.80 under real-world conditions, which is considered moderately good in terms of a diagnostic test.

Would such a procedure be worth the added cost of training and scanning dogs? The answer depends on the total cost to successfully place an assistance dog. Depending upon the vendor and the intended working role (e.g., psychiatric, mobility, hearing assistance), the cost to train a service dog may range from $20,000 to $50,000. The typical capital outlay for a nonprofit service dog organization that uses volunteer puppy raisers for the initial 12 to 18 months of the dog’s life is anywhere from $20,000 to $35,000. Thus, we can estimate the cost to raise and train a dog that does not get placed is still approximately $25,000. Conversely, there is also a cost for releasing a dog that might have become a service dog, but this may be relatively less if resources aren’t expended to train her. Any cost savings would come from identifying dogs unlikely to succeed, and releasing them from training as soon as possible.

Therefore, one should focus on the neuroimaging to identify dogs likely to fail. In a best case scenario, Table 4 suggests that 8 out of 10 dogs were correctly identified as failures (true negatives), and 4 were flagged incorrectly (false negatives). Clearly there is some cost to false negatives, but this is hard to estimate. Early identification of the 8 dogs could have saved approximately $200,000. If the cost of the MRI was $2000, then $86,000 would have been expended on all 43 dogs, for a net savings of $114,000. Would those dogs have been identified anyway? The sex + responsiveness model showed that 8 of them could have been, but at the cost of 5 more false negatives. However, the responsiveness score is assessed after the dogs are well into service training, after which they have already incurred costs. The true value of neuroimaging, then, might be realized as an early screening tool, before dogs are selected for further training. For this to occur, dogs would need to begin MRI training at 12 months of age, and scanned at 15 months, when they would normally enter service training. However, any extrapolations about the utility of such an approach will necessarily be specific to the dogs and protocols studied. We do not know whether fMRI would have utility in a different population of dogs, e.g. military working dogs.

The higher than anticipated success rate of the dogs could be due to chance, although it is also possible that the MRI-training had a positive effect on the dogs. The MRI-training teaches the dog to lie still in a noisy environment. It is possible, through the acclimation process, they learned to do this in a more relaxed and attentive state, which generalized to other skills necessary for an assistance dog.

Beyond the predictive value of the model, the relationship of brain activation in specific regions to outcome gives new insights into why some individuals might be better assistance dogs. We found a significant relationship of outcome to activity in the caudate and amygdala. There is a vast literature linking the striatum, including nucleus accumbens, to positive expectations of reward^17^,^21^,^22^,^23^,^24^. We had hypothesized such relationships based on our previous findings in dogs that related striatal activity to cues signaling rewards like food and praise^1^,^4^,^7^,^8^. Consequently, the current experiment was designed to measure the interaction between signals that designated reward and the source of the signal (familiar handler or a stranger). In this sense, a provocative condition was added to measure how the dog reacted to a stranger. Crucially, all of the dogs maintained the trained behavior of staying in the scanner. Thus there was no outward manifestation that it mattered who gave the signals. But the brain responses, especially the correlation with success or failure, suggests that it did matter.

The mean differential response in the caudate was strongly correlated with successful placement. Thus, irrespective of who gave the signals, successful dogs showed a strong caudate response. This could represent a strong motivational signal that was unaffected by the person giving it, which would make sense as trait for a good service dog. In contrast, a low caudate response might reflect a dog with a lower motivational state, and therefore less likely to perform well as a service dog.

The amygdala response, and its interaction with the face area, showed the opposite relationship to successful outcome, particularly in the stranger condition. A strong positive interaction, as observed with both high amygdala activity and DFA activity, suggests a strong response to the identity of the person giving the signals. Because amygdala activation can be seen in response to both positively and negatively valenced stimuli, its presence here can be interpreted broadly as a measure of arousal. Arousal could occur due to either excitement or anxiety, but neither would be good for a service dog. Given the negative correlation with success, this can be viewed as an undesirable reaction, but, as noted above, the reaction was not necessarily manifest in outward behavior. This suggests an area for future research, especially on the developmental influences of amygdala reactivity.

Overall, these results suggest that brain activation, especially under a provocative paradigm, may hold significant predictive value for the outcome of some types of service dog training. Although we do not know the extent to which these findings would generalize beyond the specific training protocol and dog population studied, the mechanisms identified in the caudate and amygdala activations are likely to reflect a continuum of attitudinal responses in dogs. These responses, potentially measuring motivation and arousal, could provide a window into the dog’s internal state independently of behavior.

## Additional Information

**How to cite this article**: Berns, G. S. *et al*. Functional MRI in Awake Dogs Predicts Suitability for Assistance Work. *Sci. Rep.* **7**, 43704; doi: 10.1038/srep43704 (2017).

**Publisher's note:** Springer Nature remains neutral with regard to jurisdictional claims in published maps and institutional affiliations.

## Supplementary Material

Supplementary Information

## Figures and Tables

**Table 1:** Demographics of dogs with both MRI data and outcomes.

	Breed			Total
	GLD	LAB	LGX	
Female	0	6	13	19
Male	1	2	21	24
Total	1	8	34	43

GLD =golden retriever; LAB = Labrador retriever; LGX = lab/golden cross.

**Table 2:** Outcomes of the dogs.

Service Dog	20
Skilled Companion	6
Facility Dog	4
Hearing Dog	1
PTSD Dog	2
Behavioral Release	10
TOTAL	43

**Table 3:** **Distribution of successes and failures by sex.**

	Male	Female
Fail	7	3
Pass	17	16

Sex was not a significant effect (Fisher’s exact test, P = 0.47).

**Table 4:** Confusion matrices for different models.

	Sex+Resp		Sex + mean(CD) + AMYdif + DFAdif + AMYstr X DFAstr	
	Pred Fail	Pred Pass	Pred Fail	Pred Pass
True Fail	8	2	8	2
True Pass	9	24	4	29
NPV PPV	47%	92%	67%	94%

Threshold for predicted pass/fail = 0.5. NPV = negative predictive value, PPV = positive predictive value.

**Table 5:** Results of the final logistic model.

	Coefficient	p
intercept	−0.09	0.869
sex	0.24	0.754
mean(CD)	11.77	0.001
AMYdif	2.10	0.164
DFAdif	−2.37	0.123
AMYstr X DFAstr	−12.93	0.001
